# A novel colony‐stimulating factor 1 (CSF1) translocation  involving human endogenous retroviral element in a tenosynovial giant cell tumor

**DOI:** 10.1002/gcc.23116

**Published:** 2023-01-05

**Authors:** Astrid Lipplaa, Debora Meijer, Michiel A. J. van de Sande, Hans Gelderblom, Judith V. M. G. Bovée, Hailiang Mei, Karoly Szuhai

**Affiliations:** ^1^ Department of Medical Oncology Leiden University Medical Center Leiden The Netherlands; ^2^ Department of Pathology Leiden University Medical Center Leiden The Netherlands; ^3^ Leiden Center for Computational Oncology Leiden The Netherlands; ^4^ Department of Cell and Chemical Biology Leiden University Medical Center Leiden The Netherlands; ^5^ Department of Orthopedic Surgery Leiden University Medical Center Leiden The Netherlands; ^6^ Sequencing Analysis Support Core Leiden University Medical Center Leiden The Netherlands

**Keywords:** CSF1, HERV, novel translocation, tenosynovial giant cell tumor, transcriptome sequencing

## Abstract

Tenosynovial giant cell tumors (TSGCTs) are rare tumors arising in tendons or the synoviae of joints and bursae. The localized type is benign while the diffuse type shows expansive growth leading to greater morbidity and is therefore considered locally aggressive. Typical recurrent chromosomal aberrations are found in the majority of TSCGT and the *CSF1* gene is frequently involved. In this article, we describe a newly identified gene fusion mediated by an inversion in a case of diffuse TSGCT. Multicolor‐fluorescence *in situ* hybridization (FISH) molecular karyotyping identified a pericentric inversion of chromosome 1 in 7 out of 17 analyzed cells 46,XX,inv(1)(p13.3q24.3) [7]/46,XX [10], and with interphase FISH the involvement the *CSF1* locus was detected. After performing transcriptome sequencing analysis for fusion detection, only one out of five fusion gene algorithms detected a fusion involving the *CSF1* gene product. The resulting chimera fuses a sequence from a human endogenous retrovirus (HERV) gene to *CSF1* Exon 6 on chromosome 1, abrogating the regulatory element of the 3′ untranslated region of the *CSF1* gene. This new translocation involving Exon 6 of the *CSF1* gene fused to 1q24.1, supports the hypothesis that a mutated CSF1 protein is likely to play a vital role in the pathogenesis of TSGCT. The role of the HERV partner identified as a translocation partner, however, remains unclear. Our data add to the complexity of involved translocation partners in TSGCT and point to the potential difficulty of identifying fusion partners in tumor diagnostics using transcriptome sequencing when HERV or other repeat elements are involved.

## 1 INTRODUCTION

Tenosynovial giant cell tumors (TSGCTs) are rare tumors arising in tendons and the synoviae of joints and bursae.^1^, ^2^, ^3^ Two distinct histopathologic types of TSGCT are recognized. The localized type is the most common and these benign, slow‐growing, well‐circumscribed lesions are mostly found in the digits of hands and feet.^2^ The diffuse type is more often seen in larger joints (75% in the knee), shows expansive growth leading to greater morbidity and is therefore considered locally aggressive.^3^

Malignant TSGCT is extremely rare and can occur within an existing diffuse‐type benign lesion or as a recurrence of a previously benign lesion, and is associated with high mortality due to its metastatic potential.^4^, ^5^

TSGCTs are characterized by proliferative synovium containing mononuclear cells, multinucleated giant cells, xanthoma cells, and notable collagenization.^2^ A characteristic feature of TSGCTs is that only a small percentage of cells (2%–16%) involve the neoplastic clonal population.^6^ The clonal population expresses high levels of colony‐stimulating factor 1 (CSF1), which leads to the recruitment of non‐neoplastic cells expressing the CSF1 receptor (CSF1R). This is referred to as the “landscape effect.”^6^, ^7^, ^8^

There are typical recurrent chromosomal aberrations found in the majority of TSGCT, including trisomy of chromosomes 5 and 7 and translocations involving 1p11–13. The *CSF1* gene is located at the 1p13 breakpoint. Different translocations partners have been documented, most commonly 2q35–37, 5q22–31, 11q11–12, and 8q21–22 less frequently.^6^, ^9^ The frequently detected fusion of the *CSF1* gene to the collagen 6A3 (*COL6A3*) promotor on 2q35–37 is believed to lead to deregulated expression of CSF1 through promoter swap.^6^ However, *the COL6A3‐CSF1* fusion gene specifically has only been detected in 23%–33% of TSGCT cases.^6^, ^10^ Fluorescence in situ hybridization (FISH) experiments have shown translocations involving the *CSF1* gene locus in the majority (61%–77%) of TSGCT cases^6^, ^7^, ^11^, ^12^ suggesting additional translocation partners to be involved in the pathogenesis of TSGCTs.

In this article, we describe a newly identified gene fusion mediated by an inversion in a case of diffuse‐type TSGCT using multicolor‐FISH molecular karyotyping and transcriptome sequencing. The resulting chimera protein fuses a sequence from a human endogenous retrovirus (HERV) gene to *CSF1* Exon 6 on chromosome 1 abrogating the regulatory element of the 3′ region of the *CSF1* gene. Our data add to the complexity of involved translocation partners in TSGCT and point to the potential difficulty of identifying fusion partners when HERV repeat elements are involved in tumor diagnostics using transcriptome sequencing.

## 2 MATERIALS AND METHODS

### 2.1 Patient sample

**FIGURE 1:**
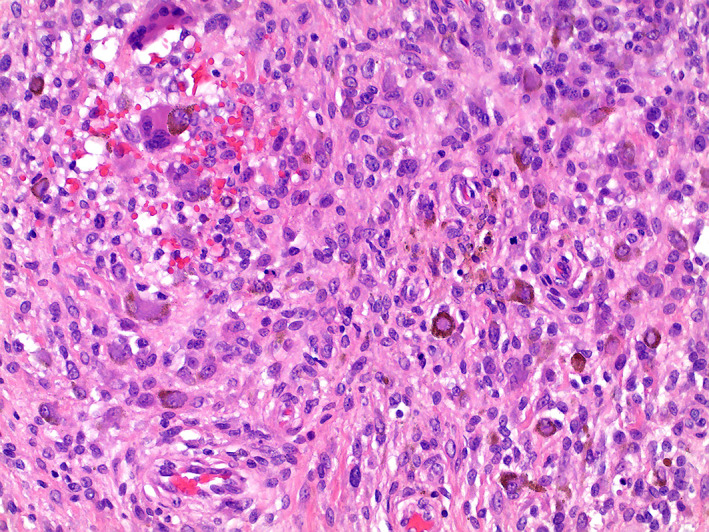
Tenosynovial giant cell tumors case L4018 hematoxylin and eosin (H&E) staining showing giant cells, iron loaded mononuclear cells, and small mononuclear cells.

The tumor sample L4018 originated from the synovium of the knee of a 53‐year‐old female and was diagnosed as a diffuse‐type TSGCT. The diagnosis was histopathologically and clinically confirmed (Figure 1). The tumor tissue sample used for this study was retrieved from the bone and soft tissue tumor biobank of the Leiden University Medical Centre (BWD005/SH/sh). The use was approved by the LUMC Ethical Review Board (B.17.039). Written informed consent was obtained from the patient.

### 2.2 Combined binary ratio labeling FISH and *CSF1* ‐split‐apart FISH

Vital cells were obtained immediately after surgery and after a short‐term culture metaphases were harvested as described before.^13^ Combined binary ratio labeling FISH (COBRA‐FISH) was performed as described by Szuhai et al.^14^ and Szuhai and Tanke.^15^

Custom‐designed split‐apart probe set bracketing the *CSF1* locus at chromosome 1p13, was designed and labeled for FISH on metaphases and tissue sections. Bacterial artificial chromosome clones: RP11‐354C7 (centromeric to *CSF1*) and RP11‐96F24 (telomeric to *CSF1*) bracketing *CSF1* locus enable the detection of translocation or inversion. Probe labeling and hybridization were performed according to previously described protocols.^11^, ^14^ Interphase FISH slide was scanned by Pannoramic 250 Flash III scanner using appropriate filters for fluorescein isothiocyanat, cyanine‐3, and 4′,6‐diamidino‐2‐phenylindole and a 40× dry objective (3DHistech, Budapest, Hungary) and visualized by the CaseViewer software (3DHistech).

### 2.3 RNA isolation‐transcriptome sequencing

Total RNA was isolated from the L4018 frozen tissue sample using a standard Trizol isolation procedure.^16^

Isolated total mRNA was subjected to transcriptome sequencing via a commercial vendor (BGI, Hong Kong) for sequencing with the HiSeq2000 platform (Illumina, San Diego).

### 2.4 RNA sequencing analysis

The sample was pre‐processed using the BioWDL RNA‐seq pipeline (version 2.0.0). First, FastQC and MultiQC were used for quality control. Then, standard Illumina adapters were trimmed using Cutadapt and the quality control was performed again. Reads were mapped against the human genome GRCh38 and Ensembl gene annotation version 94 with Hisat2 and the gene expression quantification was performed with Stringtie. The ‐B flag was additionally set in Stringtie to be able to proceed with further analysis with Ballgown in R. Furthermore, the ‐e flag was set to create a count table with raw gene and transcript counts using Stringtie's prepDE.py script. The used gene fusion tools are STAR‐Fusion (version 1.6.0),^17^ FusionMap (version 10.0.1.29),^18^ Squid (version 1.5),^19^ EricScript (version 0.5.5b),^20^ and SOAPfuse (version 1.18).^21^ Mapped bam files were visualized using IGV viewer (version 2.6.3).^22^ *CBL* mutation status was checked from transcriptome sequencing results.

### 2.5 cDNA synthesis

Total RNA from the L4018 frozen tissue sample was reverse transcribed with the Invitrogen Superscript III First‐Strand cDNA Synthesis Kit (Life Technologies, Carlsbad), according to the manufacturer's instructions using 1 μg total RNA for the reverse transcriptase reaction and oligodT primers.

### 2.6 Polymerase chain reaction (PCR) amplification

Real‐time quantitative PCR amplification of cDNA was performed to verify and analyze the *CSF1* breakpoint identified by transcriptome sequencing. Based on the RNA sequence findings, primers were designed around the putative breakpoint in the *CSF1* gene. Primers with positive PCR products are listed in Table 1. For the *CSF1‐S100A10*, we used primers published by Panagopoulos et al.^23^

**TABLE 1:** Primers used for PCR amplification and Sanger sequencing analysis.

Name	Sequence (5′ ➔ 3′)	Primer Direction	Gene	Strand	Position^a^	Chromosome band
L4018CSF1_F1	CAACTTCCTCTCAGCATCTTCT	Forward	CSF1	+	109 923 644–109 923 665	1p13
L4018_1q_RV1	GGCCTAGTATATCCCGTTTCTTC	Reverse	ERV_322034_LTR71B	+	170 773 630–170 773 652	1q24.2
L4018CSF1_F2	CAGAAGACAGACCATCCATCTG	Forward	CSF1	+	109 923 774–109 923 795	1p13
L4018_1q_RV2	AAGCTATTTGGCAGGCTAGTAA	Reverse	ERV_322034_LTR71B	+	170 773 497–170 773 518	1q24.2

^a^ Position is according to GRCh38/hg38.

The PCR amplifications were performed using the Thermo Fisher Applied Biosystems SYBR green real‐time PCR protocol, using 12.5 μl of SYBR® Green I master mix (Life Technologies, Carlsbad), 1.5 μl of the forward and reverse primers each, and 1 μl of cDNA (1:10 diluted). The PCR was run on CFX touch 96 (BIO‐RAD, Hercules) with the cycling profile of initial denaturation for 3 min at 95°C followed by 40 cycles of 15 s at 95°C, 20 s at 55°C, and 60 s at 72°C.

### 2.7 Gel electrophoresis

The PCR product was size‐separated using electrophoresis and 1% (wt/vol) agarose gel in order to confirm the presence of an amplicon of the expected size.

### 2.8 Sanger sequencing

The PCR products were submitted for Sanger sequencing (Macrogen, Applied Biosystems 96‐capillary ABI3730xl systems).

The BLAST software^24^ was used for the computer analysis of sequence data.

The HERVd database^25^, ^26^ was used as a reference database for the analysis of the sequence data.

## 3 RESULTS

### 3.1 Case description

The tumor sample originated from the synovium of the knee of a 53‐year‐old female. She first presented with pain and swelling of the right knee and was diagnosed as a diffuse‐type TSGCT. In a time frame of approximately two decades, the patient underwent nine consecutive open arthroscopies to resect the tumor and its recurrences, and she received systemic treatment with tyrosine kinase inhibitors imatinib, nilotinib, and pexidartinib without an objective radiological response. At the age of 62, she underwent total knee replacement surgery after which she became pain‐free.

### 3.2 COBRA‐FISH karyotyping and FISH

**FIGURE 2:**
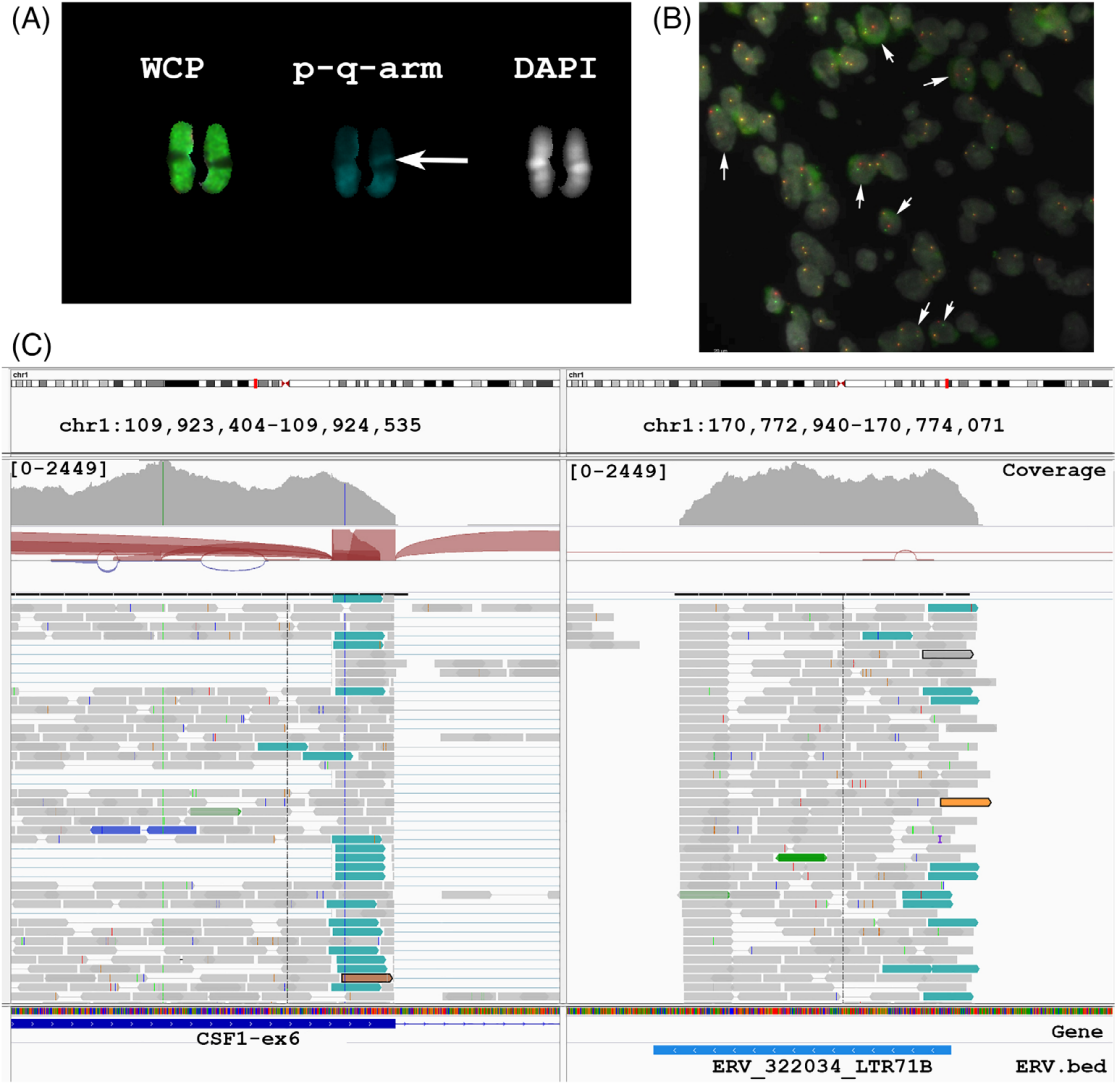
Molecular karyotyping and transcriptome sequencing results of the tenosynovial giant cell tumor (TSGCT) case L4018. (A) partial karyogram obtained after combined binary ratio labeling fluorescence in situ hybridization (COBRA FISH) analysis showing a normal and rearranged chromosome 1 left after whole‐chromosome paint, middle after short‐(p) and long‐(q) arm‐specific paint, and right after 4′,6‐diamidino‐2‐phenylindole (DAPI) staining. Arrowhead indicates the breakpoint of inv(1)(p13.3q24.3). (B) Interphase FISH using bacterial artificial chromosome probe set bracketing the *CSF1* locus. Clones: RP11‐354C7 (centromeric to *CSF1*: red) and RP11‐96F24 (telomeric to *CSF1*: green), DAPI gray. Arrowheads indicate nuclei with a split‐apart signal. (C) Transcriptome sequencing results visualized in IGV viewer. The left panel shows sequence reads covering CSF1 in the region of Exon 6, reads with discordant mate pairs (larger distance than expected) are colored in cyan and their respective mate pairs are displayed in the right panel on the long arm of chromosome 1, where no reference genes were present. The uploaded endogenous retrovirus (ERV) reference shows an almost full match with the transcribed ERV‐LTR71B. The upper part shows the exact chromosomal localizations (hg.38), coverage indicates the number of reads supporting that transcribed RNA was produced from these regions.

Multicolor FISH karyotyping identified a pericentric inversion 46,XX,inv(1)(p13.3q24.3) [7]/46,XX [10] (Figure 2A). The breakpoint was compatible with *CSF1* and break‐apart FISH probes, bracketing the *CSF1* locus showed a split signal (Figure 2B). FISH around the previously identified *S100A10* region^23^ showed no involvement of these genes.

### 3.3 RNA analysis (transcriptome sequencing, RT‐PCR)

Analysis of transcriptome sequencing for the *CSF1* fusion using five different fusion detection tools, showed that the fusion was only detected by Squid, while all other fusion detection algorithms resulted in no reported fusion gene product. Squid reported 29 potential fusion events, from which *CSF1* fusion with a gene desert observed with IGV viewer was scored the highest (>1000), other regions were not shown to be positive.

Knowing the possible involvement of the *CSF1* gene, visual inspection of the *CSF1* region in IGV showed the presence of discordant reads after paired‐end read sequencing. The sequence mate reads mapped to the chromosome 1q region, compatible with the breakpoint identified at the chromosome level. The reads, however, were not mapped to any annotated gene region, but sitting in a gene desert region (Figure 2c).

**FIGURE 3:**
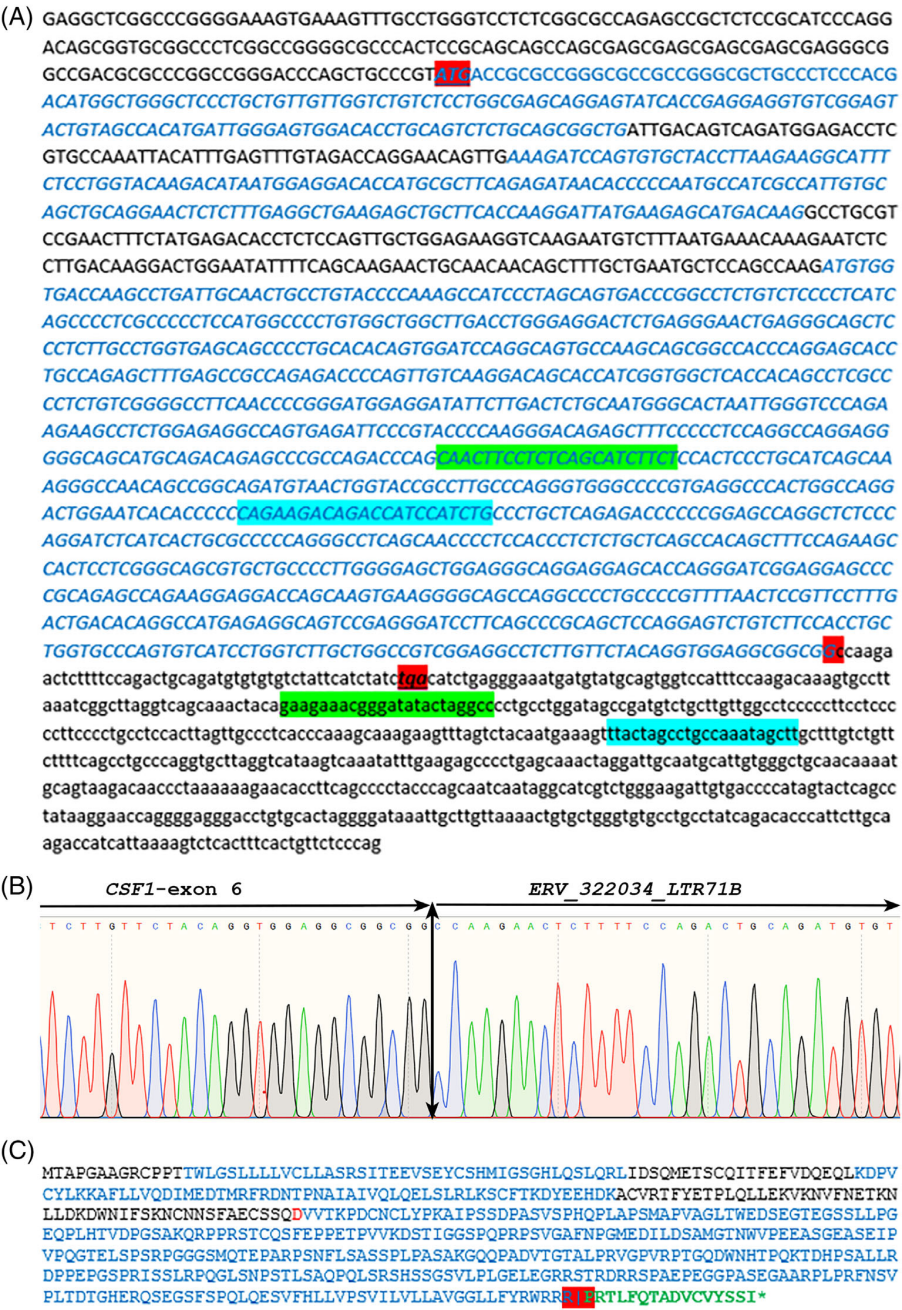
Transcribed *CSF1‐ERV‐LTR71B* fusion sequence and predicted translated protein. (A) Sequence obtained after transcriptome and Sanger sequencing. Alternating exons are highlighted in black and blue, start codon (ATG), fusion junction position and STOP codon are highlighted in red. The position of the forward CSF1_F1 and the reverse 1q_RV1 primers are highlighted in green. The position of the forward CSF1_F2 and the reverse 1q_RV2 primers are highlighted in blue. The genomic breakpoint position of the fusion junction in the CSF1 chr1: 109924190 (hg38) and the ERV‐LTR71B sequence is chr1:170773732 (hg38), respectively. The ERV‐LTR71B region is indicated as a repeat sequence in sequencing databases displayed as a small letter. (B) Partial Sanger sequence chromatogram of the cDNA amplified fragment showing the junction position (vertical arrow) of the CSF1 Exon 6 sequence with ERV‐LTR71B. (C) Predicted translated protein of the fusion product. Protein parts derived from alternating exons are highlighted in black and blue, red shows an amino acid in overlapping exons, and fusion junction position is highlighted in red. Green letters indicate the translated region for ERV‐LTR71B. ERV, endogenous retrovirus

To confirm the fusion, primers were designed to amplify the chimeric RNA after a reverse transcriptase reaction (Figure 3). A reverse transcriptase PCR analysis using primers designed around this putative breakpoint (Table 1) amplified a 703 bp and a 735 bp fragment, respectively. Subsequent Sanger sequencing revealed a translocation in Exon 6 of the CSF1 gene (nt number 109924190) fused to 1q24.2 (nt number 170773782) (hg38; Figure 3). The RNA sequencing predicted breakpoint of the *CSF1* gene was verified. In silico sequence analysis of the transcribed translocation‐partner region revealed similarities with sequences from HERV (*ERV_322034*, *LTR 71B*), that otherwise was not annotated in the reference genome.^26^ The fusion gene sequence has been deposited at NCBI with GenBank accession number: BankIt2611312 CSF1_ERV‐LTR71B; OP294996.

## 4 DISCUSSION

We identified a pericentric inversion inv(1)(p13.3q24.3) in a case of TSGCT by molecular karyotyping. Using transcriptome sequencing we identified a *CSF1* breakpoint at the 3′ of Exon 6 fused to an *HERV* element. Since this HERV translocation partner is not located in annotated genes, most of the standard fusion detection algorithms failed to report it. Only one tool we tested, Squid, is able to detect this particular event which is treated as a more general type of fusion event termed as transcriptomic structural variation.

Earlier studies showed a recurrent translocation t(1;2)(p13;q35–37) in 8 of 26 TSGCT cases.^9^ After identification of the fusion partner, *COL6A3‐CSF1* fusions were found in a significant part of TSGCT.^6^, ^7^ West et al.^6^ described TSGCT cases in which FISH analysis showed a break in the *CSF1* gene (1p13.2) in 20 out of 21 cases; however, only 3 out of 10 scorable cases involved the *COL6A3* locus. The authors hypothesize that the *COL6A3‐CSF1* fusion could bring *CSF1* under the control of a strong *COL6A3* promotor.

However, other papers questioned whether the *COL6A3‐CSF1* translocation could give rise to a functional chimeric protein leading to CSF1 overexpression since the part of the *CSF1* gene encoding for the receptor‐binding site upstream of Exon 5 is lost due to a breakpoint downstream of Exon 5.^12^, ^27^ Panagopoulos et al.^23^ identified a *CSF1‐S100A10* fusion gene carrying the translocation t(1;1)(q21;p11). This breakpoint leads to the loss of the 3′ untranslated region (3′ UTR) of *CSF1* Exon 9 that contains microRNA (miRNA) targets, a noncanonical G‐quadruplex and AU‐rich elements (AREs) controlling the expression of *CSF1*. In ovarian cancer models, different miRNA sites have been shown to downregulate the expression of *CSF1*, while binding of glyceraldehyde‐3‐phosphate dehydrogenase to the CSF1‐ARE regulates the stability and degradation of *CSF1* mRNA.^28^, ^29^, ^30^ Additionally, Woo et al.^31^ reported that interference in the miRNA target region, G‐quadruplex and AREs together substantially increased reporter RNA levels. By these mechanisms levels of functional CSF1 could increase, leading to CSF1R activation and recruitment of non‐neoplastic monocytes and differentiation into macrophages and osteoclast‐like giant cells. In a paper by Ho et al.,^12^ 39 TSGCT cases were analyzed by FISH and RNA/DNA sequencing. FISH identified *CSF1* breakpoints in 30 cases of whom 28 were confirmed by sequencing, all downstream of Exon 5 with the loss of the 3′ UTR as a consequence.

There is increasing evidence that shows the regulatory element or membrane domain loss by losing various proportions of the 3′ end of *CSF1* and new research indicates that the self‐sustaining autocrine CSF1‐CSF1R signaling loop in neoplastic cells is lacking based on single‐cell sequencing.^8^

This article further supports the pathogenesis by a change in posttranscriptional regulation of TSGCT.

Tsuda et al.,^10^ and Ho et al.,^12^ reported *CBL* mutations were identified in 35% and 29% of studied TSGCT cases, respectively. *CBL* (11q23) codes for an E3 ubiquitin‐protein ligase that is involved in cell signaling and protein ubiquitination via receptor tyrosine kinases and mutations are associated with various hematological malignancies.^32^ The binding of CSF1 to the CSF1R leads to CBL phosphorylation followed by ubiquitination, internalization, and degradation of the receptor.^33^ The *CBL* mutations could be the driving mutation in some cases of TSGCT; however, they could also merely be a secondary event. The *CSF1* fusions and *CBL* mutations in these papers were not mutually exclusive, both variations were seen in 33% of cases, which could support the hypothesis that they are not driving mutations.^10^, ^12^ Analyzing the transcriptome sequencing data, in our case, no *CBL* mutation was found.

The novel fusion event described in this article led to a novel fusion between the *CSF1* locus (1p13.2) and the 1q24.2 region on chromosome 1 in a gene desert. Blast sequence analysis of the fusion partner sequence against multiple reference databases showed similarities with sequences from a HERV. HERVs in the human genome are a heterogeneous group of viral elements that are believed to be derived from ancient germ‐line infections of exogenous retroviruses that became a fixed element in the human genome.^34^ HERVs make up ~8% of the human genome. However, due to acquired mutations over millions of years HERVs are believed to be unable to produce viral particles.^35^ Structurally, HERVs sequences are composed of *gag*, *pro*, *pol*, and *env* regions in between a long‐terminal repeat (LTR) at the 3′ and 5′. The LTRs regulate the activation and expression of HERV genes while these sequences contain main promotors, enhancers, and transactivation regions for transcription.^36^

HERV families and HERV parts can be identified using the HERVd database,^25^, ^26^ which was constructed using the output of the human genome project. The database contains extensive information on known retroviral elements found in the human genome consisting of the typical retroviral genome LTR‐gag‐pol(−env)‐LTR. Several other databases are available for detecting endogenous viral elements in the human genome, such as the Repbase and Dfam systems.^37^, ^38^, ^39^ However problems with these databases arise, for example, due to the lack of standardization in HERV nomenclature and categorization.^40^

HERVs are associated with autoimmune disease and cancer, though the precise involvement and etiology remain unclear.^34^, ^35^, ^41^ HERV overexpression has been described in many different types of cancer, like breast cancer, melanoma, lymphoma, ovarian cancer, and prostate cancer.^41^ Their neoplastic activity might be associated with the expression of HERV mRNA,^42^ functional viral proteins,^43^ or retroviral‐like particles.^44^ Furthermore, HERVs might induce neoplastic transformation through the introduction of new promotors,^41^, ^45^ activations of proto‐oncogenes^46^ or even inhibition of effective immune responses against cancer cells.^47^

For example, in some cases of prostate cancer, a translocation of the *HERV‐K_22q11*.*23* 5′ ‐LTR‐UTR sequence upstream of the transcription factor ETS translocation variant 1 (ETV1) is reported, leading to enhanced expression of the ETV1 oncogene.^48^ In B‐cell‐derived Hodgkin lymphoma cells an aberrantly activated upstream LTR promotor of the mammalian LTR retrotransposons family drives the transcription of the proto‐oncogene *CSF1*.^49^ Last, Guasch et al.^50^ report on a t(8;19)(p12;q13.3) translocation in an atypical stem‐cell myeloproliferative disorder, resulting in a *HERV‐K LTR/fibroblast growth factor receptor 1* (*FGFR1*) fusion gene.

Difficulties with the detection of these HERVs exist given their non‐coding, non‐recurrent fusions that are not identified by existing bioinformatics algorithms. Many relevant fusion events involving HERVs can be missed due to this detection gap. An example of a relevant translocation that would also be missed by regular sequencing methods is the case in a previous report by Ijzendoorn et al.,^16^ describing a translocation in the *FOS*‐gene in an epithelioid hemangioma of bone. COBRA‐FISH karyotyping revealed a balanced translocation t(3;14) in *FOS*, and further transcriptome sequencing showed a recurrent translocation breakpoint in the *FOS* gene fused to different partners in all three cases. The translocation leads to the insertion of a stop codon, leading to a truncated inactive FOS protein. This would not be picked up by next‐generation sequencing since the gene‐copy number is not altered and genes are expressed normally. Potentially, the raw data from the TSGCT case we describe in this report can be used for improving and benchmarking fusion detection algorithms that can address the limitation of gene annotation for developing better diagnostics.

In silico analysis of the *CSF1* fusion product showed an early truncation of the translated protein in the ERV element. The relevance of the finding of this new chimeric *CSF1‐HERV* fusion gene is not known, however, one might hypothesize that in this case of TSGCT the transcriptional reactivation of the associated HERV sequence may play a role in its pathogenesis. However the loss of the 3′ end of *CSF1* and abberant CSF1‐CSF1R signaling in neoplastic cells might be pathogenetically important as well.

## 5 CONCLUSION

We have described a new translocation in a case of TSGCT, involving a translocation breakpoint in Exon 6 of the *CSF1* gene fused to 1q24.2, a locus previously described as a HERV. Since *CSF1* involvement is seen in a large part of TSGCTs, a mutation in the transcribed CSF1 protein is hypothesized to play a vital role in its pathogenesis. The loss of the 3′ UTR and regulatory processes involved, previously described in TSGCT cases, could explain the CSF1 overexpression leading to neoplastic growth. The translocation partner was identified as a known HERV sequence, however, its significance in the pathogenesis is unknown. Previously reported *CSF1* fusion partners are so diverse and seem to lack any commonalities in function, therefore the role of the fusion partner could mainly lie in abrogating the negative regulatory sequence of 3′ UTR. The involvement of highly repetitive elements like ERV as driver mechanisms might be overlooked by the standard fusion reporting algorithms since the ERVs are not part of the standard gene annotation. Our results indicate the possibility of reanalysis of samples where no diagnosis has been made and a fusion event might be expected. Careful analysis of molecular data (i.e., sequencing results) is crucial in unusual cases as patients may become eligible for targeted therapy.^51^

## Data Availability

The data that support the findings of this study are available from the corresponding author upon reasonable request.
